# Report on Quackery

**Published:** 1856-10

**Authors:** Albert Smith

**Affiliations:** Peterborough, N. H.


### DEPARTMENT OF SELECTIONS.

### REPORT OF THE COMMITTEE ON QUACKERY.

BY ALBERT SMITH, M. D., PETERBOROUGH, N. H.

At the annual meeting of the New Hampshire Medical Society, June, 1855, it was

*Resolved*, That a committee of three be appointed, who shall report at the next meeting of this Society, upon the subject of Quackery, and how far it is consistent for members of the medical profession to countenance empirics by meeting them in consultation.

*Committee.*—Dr. A. Smith, G. B. Twitchell, N. Wight.

The committee consider the subject of the resolution opportune, and deem it important that we should be aware of the exact condition of our profession, and of the alarming and dangerous progress which has been made in every species of quackery. If it is right to designate this as an age of advancement, of enlightenment, it is no less true, that it is an age of charlatanry; an age in which all pretensions, all shams, all braggardisms find a marvellous currency, and a ready adoption.

There is some element in the public training, that shuts the ears of the people to science and common sense, and opens them to hollow cant, unfounded boasting and wicked pretension, that is wrong in principle and dangerous to the welfare of the community. It is liberty carried to licentiousness. It is the total subversion of the conservative principle, that should always be our safeguard against innovation not sanctioned by strong presumption of usefulness. Nothing can be more painful to the philanthropist, than to see the proclivity of the age to quackery; to behold in every corner of our land such immense quantities of nostrums, and to know that these worthless compounds are designed to be administered at random, to be taken for real as well as fancied ills, as chance or officiousness may direct; and, still more so, to be aware of the great number of quacks, of every description, who prowl upon the community in every town, and feed, like vampires, upon the blood of the people. But this trifling with human life is one of the rights of the people, with which we can not interfere; they believe it is only the fear of danger to their craft, from this source, that induces physicians to say ought against it. And so strong an antagonistic feeling has arisen, that they regard the reliance upon nostrums and quack administration of medicine more valuable than any dependence upon a learned profession. The profession to them is "pearls before swine."

It becomes an important question, in offering this report, to determine what quackery is at the present day; and to see whether any of this odious quality still adheres to those who class themselves among the regular faculty of medicine. We often hear it said of many an accomplished and scientific physician, that he has too little quackery to succeed, *i. e.* he is too honest; he scorns to practice deceit; he does not flatter the foolish whims and prejudices of the people, but looks at disease through the medium of science and common sense. It has been supposed that no medical man could be respected as a physician, without making pretensions he could never fulfil, and uttering boastful and exaggerated representations of what he has done, and is able to do. Is this so? Can the physician only sustain himself by resorting to the tricks, arts and manœuvers of the empiric? It is humiliating to think that the people are often actuated by every other sense than common sense, and seem to see as much merit in the most unblushing quack, as in the best physician; and yet, with all these apparent derelictions, we can not but think that the class is constantly increasing, who abhor every species of quackery in or out of the profession.

We believe that the straight-forward and honorable course is the best for the physician—he does not need any form of quackery to succeed, nor any submission to a vitiated and unenlightened public opinion—he requires only the necessary *tact*, by which he can only succeed in any business, and without which, he will fail in all.

It is a matter of regret, that many physicians think a little manœuvering and well practiced deceit very necessary to their reputations. We find some of this class always having severe and alarming cases of the most dangerous and rare diseases, and, on all occasions, patients just hanging over the brink of the grave, and yet all getting well, to their great honor and skill. These men seem to live in a medical atmosphere, always using the most recondite terms in medicine, and the most unusual and unintelligible names of disease, to show off their learning and extraordinary attainments, by which you could only infer that great skill must certainly inhere when there was such an amount of *occult* erudition.

It is no less reprehensible that many physicians of our times yield their assent to the erroneous and unintelligible doctrines of the humoral pathology, and thereby give continued currency to this great medical delusion of our fathers and of the age just past. It is thought to be dangerous to one's reputation to demur to this popular pathology, to scout at it as a figment of the brain, as a mere play upon words. It is very probable, if you discard this popular delusion, that many self-conceited and self-complacent people, who know so much about medicine, (and who does not understand medicine?) will desert you for your heresy, and go where they can get such talk as they deem reasonable and agreeable to the universal belief of the community. Now the quack, who only sustains himself by sham doctrines, understands the people—he flatters them with his good opinion of their intelligence and acuteness—he adopts their pathology, and thus easily satisfies and stultifies his admiring and wondering patrons. They can not see why physicians may not explain disease with equal facility. It is distasteful to have them go into a scientific examination of their cases—the modern means of exploration of the chest, and other scientific methods of investigation are unmeaning and unsatisfactory to them—they prefer to have the Gordian knot cut at once, and to have the talismanic words of humors and impurity of the blood, solve every difficulty. They scout the idea of any labor in investigating disease; they believe that this knowledge comes by intuition. The great empiric needs only to look at the face or in the eye of his dupe, and his diagnosis is made. It is then detailed in such language as his followers are supposed to understand.

This leads me to remark here, whether we do not, often undesignedly, make a show of empiricism, by presuming that the people understand us, when we talk upon medical subjects, which are so very familiar and simple to ourselves. It is one of the most desirable acquisitions to be able so to explain disease, in plain and intelligible language, that it shall be clear and convincing to the common mind. When, as a profession, we can do this, that day marks the decline of this miserable pathology that has hung for ages, like an incubus, upon the world.

This pathology, though considered extinct by the profession, yet holds great sway in the popular mind;—and no less absurd and unscientific is the prevalent nursery doctrines of *invermination* as a cause of disease in infantile life But no man will fall into the popular belief that such a thing as *worm fever* or *worm fits* are of very common occurrence; this is bad enough for old women and quacks, and yet, I fear that all our profession are not beyond it. It is a ready solution of a point of pathology, sometimes exceedingly difficult to settle, and, beside always being acceptable, it often saves a man the exhibition of much poor physiology and pathology. But no practitioner, who makes any pretensions to science, can for a moment adopt them—he can see nothing in them to aid him in his diagnosis. If the case admits of an easy diagnosis, or there are points of obscurity in it, how can he make these known to his patient? We do not think there is any difficulty for the practitioner who really understands a case, and has a common use of his vernacular tongue, to express himself in such plain and simple language as to make himself perfectly understood. Of course, he will avoid technical phrases—terms having only a medical meaning—and all expressions that would seem pedantic or obscure. In this manner, if the diagnosis is made out, or there is much about it yet obscure, all this may be made intelligible to most minds.

If there is any one thing, beyond all others, that distinguishes the profession of our times, from that of by-gone days, it is that we have discarded, in our intercourse with our patients, all the unmeaning terms, the fanciful and jaw-breaking names, all the little mysteries and secrecies that rendered the art somewhat occult with our fathers, and have substituted for it plain common sense—and this has quite lowered us in public estimation;—and though we stand as knowing vastly more than our predecessors, having made great advances in every department of medical science, yet we are really in less repute with the people than the unblushing, boasting, presumptious quack.

What should be the treatment of quackery? It should be that of abomination, loathing and hate. It should be considered the unclean thing—foul to the touch, wicked and treacherous to the soul—as a deadly miasm to every generous and benevolent emotion—as the death of every upright principle.

We may hold it almost a self-evident maxim, that there is some obliquity in that mind which is carried away with one or any of the forms of empiricism—an inability to be governed by right opinions and sound principles. We do not see how it can be consistent with our principles to have the least intercourse with any form of empiricism, or hold any consultations with empirics; for how can we tolerate their ignorance, baseness or folly, or if they are not really so ignorant as they are vicious, how can we endure their base betrayal and prostition of our noble profession?

The prevalence of the great number of pseudo theories in the healing art, the singular tendency of the age to medicine, and the unwavering reliance upon its therapeutic effects, are among the peculiarities and dangers of our times.

Medicine is a dangerous instrument to be thus dallied with:—and, independent of its demoralizing effect upon the mind, when embraced in any of the systems of charlatanry, it is fraught with the greatest danger to the body. An untold catalogue of evils, arising from this source, could no doubt be found, if we only had power to trace out, accurately, the history and effects of the one hundredth part of the nostrums taken; very much of the ill health, arising from the shattered condition of the nervous system, prostration of the digestive and assimulating processes, &c., often owe their entire origin to this blind and indiscriminate medication. It would seem bad enough to take medicine, when it is absolutely necessary, and when it is judiciously prescribed by those who really know with what intent it should be administered; and yet, how little reliance is placed upon those educated to the business, and how much upon him, who indiscriminately promises a cure because he does not know of any danger. It is thought among the people, untrained and undisciplined in thought or study, that nothing is easier than to prescribe medicine or to understand a medical theory—also to judge of the qualifications of a physician, whether he is scientific or not. How self-complacently do many of the female followers of Hahnemann berate the poor benighted disciples of true medicine, as laboring and striving in the fog, when such a pellucid and etherial atmosphere as homœopathy is all around them. How do they pity his delusion, that he can not see that the increase of power in medicine is in direct ratio to the diminution of its quantity—that attenuation is the law of nature—when he, poor soul, would say that it was the law of fools. It would seem that the greater the absurdity, the wider the currency, and that nothing could be too monstrous for belief.

It is the opinion of your committee, that it should be the established rule of every physician to have nothing to do with irregular or unqualified practitioners. A proper self-respect forbids it, as well as the honor of our time-hallowed calling. If we meet such persons and consult with them, we give our sanction to their errors, and give them currency and standing in the community. With most of those classes of quacks, such as the itinerant Indian doctor, the mesmeric or spiritual doctor, the botanic, herb and water-cure doctor, and a herd of other personifications of ignorance, rascality and impudence, you have no trouble in declining all intercourse. The people do not ask us to degrade ourselves to their level. It is, however, somewhat different with homœopathy—this comes in a genteel guise, and in its bearing, it occupies the highest niche in the temple of empiricism. Their followers make great pretensions to knowledge, for they profess to go on with us in all the preparatory course of a medical education, till we separate, world-wide, on materia medica and therapeutics. Here come up their wild theories and strange doctrines, and such an amount of nonsense and inconsistency as was never met with before among reasonable and sane men.

In their efforts to appear learned, one is forcibly reminded of the description given of Sir Hudibras' learning.

"But when he pleased to shout, his speech,
In loftiness of sound, was rich;
A Babylonish dialect,
Which learned pedants much affect:
It was a party colored dress
Of patch and piebald languages;
'Twas English cut on Greek and Latin,
Like fustian heretofore on satin;
It had an odd, promiscuous tone,
As if he'd talked three parts in one;
Which made some think, when he did gabble,
Th' had heard three laborers of Babel,
Or Cerberus himself pronounce
A leash of languages at once."

With their untenable and absurd theories, they pretend to accomplish, with infinitesimals, attenuated to inconceivable nothingness, as much as we can by the administration of medicine; while their whole reliance is upon *nature* to restore healthy action, they carefully removing every possible hindrance. Can we fellowship this delusion? Never, till the most incongruous and dissimilar things in nature coalesce and unite.

There is one thing more we must not omit to mention, which if it is not *bona fide* quackery, it is nearly as bad in baseness and meanness. It has not been an uncommon sight, heretofore, when Thompsonianism was in its zenith, for some of the regular practitioners, who thought they could make some capital out of the popular prejudice against *calomel*, to countenance this delusion, and pretend to be converts, in part, to this system, by discarding this useful remedy and adopting some of the treatment.

In our times, when lobelia and steam have passed away, and new-vamped schemes of medicine have come up among the people—who know so much about medicine—many physicians (be it said with shame,) in their efforts to retain the patronage of those affected with these delusions, give themselves up entirely to these absurdities, or attempt partially to engraft on the profession these new-fangled notions, to the great injury of their honesty and integrity.

A regular practitioner, tainted with these follies, may occasionally be heard to sneer at the old school, and be seen dispensing hydropathy to its disciples, and the old regime to others; or dealing in little pills and globuli to his homœpathic adherents, and the grosser treatment to his old friends, who know not nor want anything better than the old profession.

Some become partial converts to the mesmeric and spiritual delusion—they permit a few isolated facts alone to lead them from the truth, till they nearly become unable to appreciate it amid the maze of error in which they become involved. Their respectable friends become deluded, and they have not nerve enough to denounce its follies and extravagancies.

They hope by this course to retain the good opinion of such persons, but they are, I fear, too much like the dog in the fable, who dropped the piece of meat in its mouth to seize its shadow. Nothing like integrity will serve the best interests of the physician, and nothing, in the long run, will sustain him and help him forward, as the sturdy maintenance of his profession in all its dignity and honor.

This prostitution of the profession, to subserve what has been deemed the pecuniary interest of the physician, is only equalled by another gross imposition, that of permitting the people, because our support comes from them, to exercise toward us an overbearing spirit—to lord it over us as though they had the making and unmaking of our reputations—to dictate to us what opinions we should hold in medicine, or even what we should believe on other subjects! How common is it, to hear it said that the physician must not be a party man—he must not be too much committed to any sect or denomination in religion—and he must be careful that he does not take too much interest in local matters—he may injure his popularity. Shall we yield to these unreasonable and unjust requisitions? No, unless we wish to incur the contempt of these very persons who would thus degrade us. When we have opinions and convictions which we are sure are right, no fear of obloquy or dislike should, on all proper occasions, prevent us from openly and boldly avowing them. We hold in detestation that man, whose whole life is only a system of compliances with the popular will, whatever it may be. He knows no integrity, no principles, but to keep his sails always trimmed to the popular gale.

Do our patrons not honor us by giving us the preference in employing us, and should we not be grateful for this? But they expect an equivalent and more than an equivalent, and this is duly rendered. But is not this same honor rendered to the veriest quack as well as to ourselves, and do not these render immense satisfaction often, when we cannot or will not? Do they not solve all doubts, make favorable prognoses, and dispel all fear of danger, when we pause and hesitate?

We should always be grateful for any honest support we may receive, or any just appreciation of our professional attainments, and yet we cannot afford to be so disinterested as to receive the *honor* as our only remuneration; we must have something more substantial, and even then, the obligation is not on our part.

We fear that the honor of our profession is not properly sustained—that there is too much truckling to a vitiated public opinion—too much subserviency to the popular will—too much submission of great and well-defined principles to the crude and half developed schemes of the ignorant and unprincipled quack.

Your Committee have thus decided, that we can have no intercourse with quackery in any "way, shape or manner,"—that it is not honest nor honorable to do so. They would impress it upon the minds of all, that in every way and manner, physicians should stand for the truth and honor of the profession. But the road to wealth is through empiricism—it is the golden course—it yields great gains; yet it should be shunned, as a syren's voice, that lures to destruction. Its paths cannot be entered without defilement; it breaks down the man and substitutes an abortion, a miserable sham. We desire not to see a poorer piece of humanity—of prostituted manhood, than is seen in the conversion of a regular, well educated physician, to the ranks of any of these popular theories of medicine.

Far better is it to *starve*, with our integrity and principles in a sound and healthful condition, than at the expense of an uneasy, restless state of violated honor, to be crowned with the greatest human prosperity.

